# Delayed Onset of Spinal Anesthesia in a Chronic Opioid User Undergoing Cesarean Section

**DOI:** 10.7759/cureus.79247

**Published:** 2025-02-18

**Authors:** Michael Gross, Quincy Saint-Hilaire, Garrison Kohler, Imani Thornton

**Affiliations:** 1 Anesthesiology, HCA Florida Westside Hospital, Plantation, USA; 2 Medical School, Nova Southeastern University Dr. Kiran C. Patel College of Osteopathic Medicine, Davie, USA; 3 Medical School, St. George's University School of Medicine, St. George, GRD; 4 Anesthesiology and Critical Care, HCA Florida Westside Hospital, Plantation, USA

**Keywords:** neuraxial analgesia, neuraxial labor analgesia, obstetric anesthesia and analgesia, obstetric anesthesiology, opioid use disorders, prenatal opioid use

## Abstract

Delayed onset of spinal anesthesia can pose challenges during cesarean section (CS) in patients with a history of chronic opioid use. We present a case of delayed onset of spinal anesthesia in a patient with chronic opioid use undergoing CS, highlighting the clinical implications and management strategies. Delayed onset of spinal anesthesia in chronic opioid users can result from neurophysiological adaptations and pharmacokinetic interactions in the central nervous system. Previous studies have suggested chronic opioid users display a delayed onset and shorter duration of action of neuraxial blockade. This can have great implications in pregnant women who require anesthesia for CS. Delayed onset can lead to misconception of a failed spinal block and conversion to general anesthesia. In reality, additional time may be needed for the block to rise to an adequate level. Additionally, delayed time to incision can lead to poor outcomes for the mother and baby in urgent scenarios. When providing anesthesia for chronic opioid users undergoing CS, it is important to understand the possibility of delayed onset of neuraxial blockade which can impact patient care.

## Introduction

The administration of spinal anesthesia stands as a foundation in modern obstetric practice as it offers swift and reliable pain relief with minimal risk to both mother and child. Spinal anesthesia is a form of neuraxial anesthesia, in which local anesthetics are injected into the subarachnoid space, surrounding the spinal cord and the associated nerve roots [1]. As a result, afferent nerve fibers are blocked causing loss of sensation, in addition to efferent motor and autonomic nerve fibers causing loss of muscle tone and sympathetic tone [1].

Like all medical procedures, complications may arise, particularly when patients have complex medical backgrounds. Common adverse outcomes include, but are not limited to, hypotension, diaphragm paralysis, hematoma, infection, nerve damage, and post-dural puncture headache [2]. Several cases of complete failure of spinal anesthesia have been documented among opioid-dependent patients [3]. A prominent challenge is the delayed onset of spinal anesthesia, a phenomenon that can significantly impact the safety and efficacy of cesarean section (CS) procedures, especially in patients with a history of chronic opioid use. A study from the Egyptian Journal of Anesthesia indicated that the incidence of failure of intrathecal anesthesia seemed to be higher in opioid-dependent patients, and there was a slower onset and decreased duration of both sensory and motor blocks [3]. This is significant because it can delay the delivery of the fetus which may not be tolerated in an urgent CS, necessitate additional analgesic options if the operation outlasts the duration of action of the spinal blockade, or prompt a conversion to general anesthesia for the operation.

The escalation of opioid use in pregnancy mirrors the broader epidemic seen in the general population [4]. Opioid abuse has been identified as a major risk factor for pregnancy-associated deaths and neonatal abstinence syndrome (defined as withdrawal symptoms in neonates who were exposed to illicit substances in utero) in various maternal mortality reviews [5]. Treating opioid dependence with opioid agonists such as buprenorphine or methadone during pregnancy helps prevent withdrawal symptoms and relapse. Meta-analysis studies have shown that the risks of birth defects associated with these medications are minimal and analogous to those in the general population [6]. Conversely, opioid abuse during pregnancy is associated with lack of prenatal care, fetal growth restriction, placental complications, preterm labor, and even fetal death. With the rise of opioid use in pregnancy, anesthesiologists should be aware of the clinical implications during obstetric anesthesia care such as decreased pain tolerance and increased anesthetic requirements due to repeated chronic use of opioid agonists. Although the effectiveness of spinal anesthesia can be complicated in patients with chronic opioid use, its implementation in obstetric anesthesia can decrease the need for postpartum opioid use [7].

## Case presentation

A 37-year-old pregnant female G3P2002 at 39 weeks and one day gestation presented for an elective primary caesarian section due to a history of third- and fourth-degree lacerations in previous vaginal deliveries. She had a past medical history of bipolar disorder, seizure disorder due to previous traumatic brain injury, chronic lumbar and cervical back pain due to previous motor vehicle accidents, and a history of opioid abuse. She endorsed former intravenous heroin use and was actively taking buprenorphine 8 mg sublingual once daily. She last took buprenorphine the day prior to presentation. Her other daily medications included Ativan, baclofen, Bentyl, Cymbalta, Keppra, and Seroquel. She was 5 feet 10 inches tall, weighed 257 lbs (BMI 37.3 kg/m²), and all of her vital signs were within normal limits. She had no signs of acute infections nor spinal abnormalities. She had normal strength and sensation in her lower extremities. Her airway examination appeared adequate with a Mallampati score of 2, good dentition, and normal range of motion of her neck. Her preoperative labs were significant for a hemoglobin of 7.5 (Table 1) and two units of cross-matched packed red blood cells were available and on hold due to her pre-existing anemia and increased risk of bleeding in a repeat CS.

**Table 1:** Preoperative CBC results WBC: white blood cells; RBC: red blood cells; HGB: hemoglobin; HCT: hematocrit; MCV: mean corpuscular volume; MCHC: mean corpuscular hemoglobin concentration; RDW: red cell distribution width; PLT: platelet; MPV: mean platelet volume; L: low; H: high; HC: critical high

Lab value (units)	Result	Reference range
WBC (cells per 10^3/uL)	6.2	4.0-10.5
RBC (cells per 10^6/uL)	3.57 L	3.93-5.22
HGB (g/dL)	7.5 L	11.2-15.7
HCT (%)	26.0 L	34.1-44.9
MCV (fl)	72.8 L	79.0-94.8
MCHC (g/dL)	28.8 L	32.2-35.5
RDW (%)	16.1 H	11.7-14.4
PLT count (cells per 10^3/uL)	172	150-400
MPV (fl)	9.8	9.4-12.3
Absolute neutrophils (cells per 10^3/uL)	4.26	1.56-6.13
Segmented neutrophils (%)	69.2	34.0-71.1
Lymphocytes (%)	22	19.3-51.7
Monocytes (%)	5.9	4.7-12.5
Eosinophils (cells per 10^3/uL)	1.8	0.7-5.8

With the patient in the seated position, a spinal was performed at the L3-L4 space with a midline approach using a 24-gauge Pencan needle with a guiding needle. CSF was aspirated which confirmed the spinal needle was within the intrathecal space. A single injection was administered containing 1.7 ml of bupivacaine 0.75% with dextrose 87.5 mg/ml, 25 mcg of fentanyl, and 0.3 mg of morphine preservative free. No injectate was misplaced during the procedure and CSF was aspirated halfway through injection to confirm continued intrathecal injection. The spinal was performed without complication and the patient was immediately repositioned into supine position.

Within the first five minutes thereafter, the patient became hypotensive as often anticipated after spinal anesthesia. Phenylephrine was given to support her blood pressure, followed by ephedrine due to continued hypotension and reflex bradycardia after phenylephrine was given. After 10 minutes, she reported tingling in her legs but denied complete loss of sensation and she could still raise both legs. Her level of sensory block was checked and determined to be at the L2 level. The level of blockade was checked again several minutes later which showed that it had risen a few inches higher bilaterally. The patient denied pain, but she voiced concern that the block was not going to work. Since it was still slowly rising and she and the baby were stable, we reassured her that we would continue to monitor it as it rises before concluding it as a failed spinal. The level of blockade was monitored every five minutes until she had a satisfactory blockade up to the T4 level approximately 35 minutes after the spinal. Fetal heart tones remained reassuring after the spinal. Sufficient analgesia was confirmed by the surgeon prior to incision with an Allis clamp by gently clamping the patient’s skin without any patient discomfort, and the surgery was completed without complications or patient discomfort. Postoperatively, the patient remained stable. She began to complain of pain within an hour after completion of the surgery which was effectively managed by the pain management team. She and the baby were discharged from the hospital on postoperative day three in good condition.

## Discussion

Spinal anesthesia provides analgesia to the patient and facilitates a safe delivery via CS with many advantages, including a high safety profile, improved analgesia, and quick onset and offset of action. Several studies have displayed that neuraxial anesthesia may be altered in chronic opioid users. Opioid-dependent patients are prone to reduced duration of action of spinal anesthesia and a lower level of sensory block [8-11]. Derakhshan et al. reported a delayed onset of sensory block in chronic opioid users undergoing lower extremity surgery with spinal anesthesia. In opioid addicts compared to non-opioid addicts, the authors reported nearly a two-fold increase in the onset time needed to achieve a T10 dermatome sensory block (5.72 minutes vs. 3.16 minutes, P<0.001) [12]. Most literature discussing the effects of chronic opioid use on local anesthetics has primarily studied patients undergoing lower extremity surgeries or non-obstetric lower abdominal surgeries. This case in particular brings to consideration a different subset of patients who are at risk for delayed onset of action of neuraxial anesthesia.

The level of sensory block needed for CS is higher than for those undergoing lower extremity or non-obstetric lower abdominal surgeries. Although somatic pain from the skin and abdominal wall incision during a CS can be eliminated with a level of block to the T10 dermatome, a level as high as T4 is needed to control visceral pain originating from the uterine incision and manipulation [13]. Because there are many factors that can affect the speed of onset of spinal anesthesia, the average time of onset has not been well documented. However, several review articles report that a maximum of 15-20 minutes should be allowed before considering a failed block [14,15]. In this case, we observed a 35-minute onset time to achieve a level of T4. Since this was not an urgent or emergent case, we were able to wait and ultimately proceed with a successful block.

Previous research has demonstrated an increased incidence of failed spinal anesthesia in patients with chronic opioid dependence. In the study by Youssef and Abdelnaim, there was a delay in onset time of anesthetic after administration of spinal anesthesia found in 33% of patients suffering from drug addiction, compared to 4% of patients who did not [3]. Depending on the urgency of the CS, anesthesia providers may need to abort a spinal with slow rise of sensory block and convert to general anesthesia. This may be one factor contributing to the increased incidence of “failed spinal anesthesia” in chronic opioid users. Youssef et al. also report a higher incidence of hypotension and nausea in opioid-dependent patients than in non-opioid dependents [3]. Hypotension may present more often in opioid-dependent patients due to increased risk of dehydration, vomiting, and sympathetic block. In our case, the patient quickly developed hypotension despite the delayed rise of sensory block.

While the correlation between chronic opioid use and delayed onset of spinal anesthesia is frequently cited in the literature, the mechanism remains poorly understood. One preclinical study has demonstrated reduced potency of lidocaine in a dose-dependent and time-dependent manner by repeated morphine injections in rats [16]. A cross-tolerance between opioids and local anesthetics may be due to an opioid-induced alteration in the expression of voltage-gated sodium channels where local anesthetics act [16,17]. An additional explanation could be the release of mediators in the dorsal horn capable of acting on primary afferents to cause tolerance not only to opiates but also to local anesthetics [16]. Further research is ongoing to study the mechanism of opioid-induced tolerance to local anesthetics.

Overall, these multifaceted effects highlight the intricacy of anesthesia management in individuals with opioid use disorder. With regular use of opioids our bodies build a tolerance because of the desensitization of opioid receptors. This decrease in response to identical doses of medication extends to the receptors. Despite our numerous advancements in anesthetic medications and techniques, the administration of neuraxial anesthesia in patients with chronic opioid use remains challenging. One of the goals of our case study is to promote the necessity for broadened research on the mechanisms causing delayed onset of spinal anesthesia in this population. Additional studies should specifically target obstetric patients. Understanding the underlying mechanisms will allow effective management strategies and optimize outcomes in both mothers and their infants.

Our case study proposes key identifications of some difficulties of managing spinal anesthesia, particularly in parturient chronic opioid users undergoing CS. It is important for anesthesiologists to be aware of the potential for variations in drug response and pharmacodynamics in this population. Based on our observations and the current literature, we advocate for several recommendations to optimize anesthesia management in these patients. First, anesthesiologists will need to conduct thorough patient evaluation and planning. The preoperative assessment should include a detailed medical history with consideration of opioid dependence. Secondly, we recommend that anesthesiologists practice heightened supervision of sensory blockade progression in complicated patients, making adjustments as necessary to ensure adequate analgesic coverage. Thirdly, the use of adjunctive medications to enhance the efficacy and duration of spinal anesthesia in opioid-dependent patients should be considered. In such a scenario, anesthesiologists must carefully assess both the benefits and risks associated with each medication when tailoring their usage. Adjuvants, such as central opioid receptor agonists like fentanyl and morphine, alpha-2 adrenergic agonists, and steroids can further strengthen the efficacy and duration of neuraxial anesthesia [18]. These medications may offer improved pain relief and potentially reduce the need for supplemental analgesia postoperatively. Safari et al. found that intrathecal dexmedetomidine as an additive to spinal anesthesia significantly reduced the onset time and prolonged the duration of sensory block in chronic opioid users [19]. However, the potential for dexmedetomidine to induce hypotension and bradycardia must be noted. Similarly, while opioids provide a synergistic effect, they can also further decrease respiratory drive and worsen nausea, vomiting and pruritus, especially in opioid-dependent individuals [18].

Lastly, we recommend enhanced awareness of time sensitivity and flexibility. Urgent vs. non-urgent deliveries provide two separate approaches. During non-urgent deliveries such as our case study, anesthesiologists may choose to wait for the spinal block to take full effect, which could mitigate the need to convert to general anesthesia and improve outcomes and patient satisfaction. For many patients, it is important to be awake and bond with their newborn immediately after delivery. An abrupt conversion to general anesthesia would take away that possibility and could be very unsettling to the mother without proper counseling and shared decision-making. While in an urgent CS, anesthesiologists should know the risks for delayed onset of spinal anesthesia and discuss with the obstetrician when deciding between spinal vs. general anesthesia. There is a broad spectrum of urgency in certain scenarios which may or may not allow time for a spinal when there is a risk for delayed onset of sensory block. We encourage all anesthesia providers to be aware of this risk and discuss it with obstetricians and patients in order to make a well-informed anesthetic plan and counsel the patient on all possibilities.

## Conclusions

This case report highlights the anesthetic implications in pregnant patients with a history of chronic opioid use undergoing CS. The onset of a satisfactory sensory block in these patients can be significantly delayed compared to non-chronic opioid users which would delay the time to incision and ultimately the time to delivery. The potential mechanisms discussed in the literature are opioid-induced alteration in the expression of voltage-gated sodium channels where local anesthetics act, and a release of mediators in the dorsal horn capable of acting on primary afferents to cause tolerance not only to opiates, but also to local anesthetics. Further research is ongoing to study the mechanism of opioid-induced tolerance to local anesthetics. There is evidence that intrathecal adjuvants may increase the speed of onset and improve the quality of the block. The mother and fetal history and condition must be considered along with the time sensitivity of the surgery when deciding on an anesthetic plan.
